# Chemical Load-Induced Surface Nanocrystallization in Nitrided Martensitic Stainless Steel

**DOI:** 10.3390/nano16030151

**Published:** 2026-01-23

**Authors:** Xu Yang, Honglong Che, Mingkai Lei

**Affiliations:** Surface Engineering Laboratory, School of Materials Science and Engineering, Dalian University of Technology, Dalian 116024, China; yx0727@mail.dlut.edu.cn (X.Y.); chehl@dlut.edu.cn (H.C.)

**Keywords:** nitriding, expanded austenite transformation, nanocrystallization, TEM

## Abstract

Surface nanocrystallization is a critical approach for improving mechanical and functional properties of materials. Beyond conventional mechanical routes, chemical loading presents a promising pathway for nanocrystallization via interstitial-driven phase transformation. However, the characteristics and mechanisms underlying chemical load-induced nanostructuring remain insufficiently elucidated. This work investigates the surface nanocrystallization of 17-4 PH martensitic stainless steel during low-temperature plasma nitriding at 350 °C. Microstructural characterization combining XRD, EPMA, and TEM revealed a nitrogen-saturated layer with a maximum hardness of 13.5 GPa. The modified layer consists of nanoscale domains formed via a diffusionless martensite-to-austenite transformation, as evidenced by broadened FCC peaks, dark-field images, and the absence of elemental partitioning in EDX maps. This process is driven by the cyclic accumulation of chemical and elastic-strain energy at the advancing nitrogen diffusion front, triggering a self-sustaining, periodic transformation. This study introduces a chemical-driven nanocrystallization mechanism for novel design of surface-nanostructured steels via controlled thermochemical processing.

## 1. Introduction

Nanocrystallization refers to processes that yield materials with grain structures constrained to the nanometer scale. The consequent high density of grain and phase boundaries profoundly modifies a material’s physical, chemical, and mechanical properties. Following Gleiter’s pioneering contributions, this field has grown into a major research domain within materials science [1,2]. Established nanocrystallization strategies are fundamentally categorized by formation mechanisms into bottom-up and top-down approaches [3]. Bottom-up methods assemble nanostructures from atomic, molecular, or ionic precursors. Techniques such as chemical vapor deposition (CVD), sputter deposition, and electroplating are prominent examples, where precise control over processing parameters enables the tailored synthesis of nanocrystalline materials with specific properties [4]. In contrast, top-down approaches commence with bulk solids and diminish their structural scale through severe plastic deformation. Techniques, including equal channel angular pressing (ECAP) and ultrasonic shot peening (USSP), refine grains to the nanoscale by accumulating high-density dislocations and ultimately forming new grain boundaries [5]. This methodology is broadly applicable to metallic systems, intrinsically altering their mechanical and physical properties [6].

While mechanical loading is the most common driver for top-down refinement, external stimuli such as thermal or chemical loads can also initiate nanocrystallization. Thermally activated processes, for instance, can produce uniform multiphase nanocomposites through precipitation during heat treatment [7]. Repeated martensitic transformation under cyclic thermal loading likewise leads to progressive grain refinement [8]. Chemical loading presents an analogous pathway. Introducing foreign components into a metastable solid solution generates internal chemical potential and stress/strain fields comparable to those produced by thermal or mechanical means. This can induce localized structural instability and nanoscale decomposition [9]. For example, nitrogen supersaturation during low-temperature nitriding of austenitic stainless steel induces significant lattice expansion. Stress relaxation via dislocation slip creates a nanostructure characterized by high-density, nanoperiodic stacking faults [10]. While not conventional nanocrystallization, this diffusionless transformation yields nanoscale hexagonal close-packed (HCP) domains delineated by specific stacking sequences.

The present study systematically investigates the nanocrystallization behavior and underlying mechanisms in a low-temperature nitriding-treated 17-4 PH martensitic stainless steel. This work not only advances the fundamental understanding of surface nanocrystallization mechanisms in martensitic steels but also offers a practical experimental and theoretical basis for designing high-performance surface-nanostructured materials via controlled thermochemical processing.

## 2. Materials and Methods

The commercial 17-4 martensitic stainless steel was used in the nitriding experiment. The chemical composition of the 17-4PH martensitic stainless steel used in this study is provided in Table 1. The material was subjected to a solution treatment at 1040 °C for 2 h under a protective atmosphere to prevent oxidation, followed by quenching in oil to achieve a supersaturated martensitic structure. Subsequently, aging treatment was conducted at 595 °C for 4 h to promote precipitation hardening, after which the samples were furnace-cooled to room temperature.

Plasma nitriding was carried out using a plasma-based low-energy ion implantation (PBLEII) system [11]. The key components consisted of a 2.45 GHz microwave-powered electron cyclotron resonance (ECR) plasma source and a sample holder equipped with an auxiliary heater and a pulsed negative bias power supply. During the nitriding process, the system was evacuated to a base pressure of 1.5 × 10^−3^ Pa, and then nitrogen gas was introduced into the plasma source chamber until the pressure reached 5 × 10^−2^ Pa. After the pressure had stabilized, plasma discharge was initiated by applying microwave power. The auxiliary-heated samples were connected to a pulsed negative bias of −2 kV, and the process temperature was maintained at 350 °C. The primary mass transfer mechanism involved low-energy ion implantation accompanied by simultaneous diffusion into the substrate. The total fluence of nitrogen ions was calculated on the basis of a constant average ion current density of 0.8 mA/cm^2^ over a treatment period of 4 h.

To reveal the microstructural characteristics, the nitrided specimens were etched with a reagent composed of 10 g of FeCl_3_, 10 mL of HCl (analytical grade), and 120 mL of deionized water for approximately 10 s. The resultant microstructure was inspected using a light-optical microscope (LOM, DMi8, Leica Microsystems, Germany). Nitrogen concentration-depth profiles were obtained by field emission electron probe microanalysis (EPMA, JXA-8530F PLUS, JEOL Ltd., Japan), operating at an accelerating voltage of 15 kV and a beam current of 50 nA. Hardness of the nitrided layer surface and cross-section was measured using an HXD-1000TM Vickers microhardness tester with a load of 0.25 N. Phase identification was carried out using X-ray diffraction (XRD, Empyrean, Malvern Panalytical Ltd., The Netherland) employing Cu–Kα radiation (λ = 0.15405 nm) with a step size of 0.02°. Cross-sectional TEM specimens of the nitrided samples were fabricated on a Dual-Beam-Focused Ion Beam system (Helios G4 UX, FEI Company, Hillsboro, OR, USA) from the near-surface region of the nitrided layer. Cross-direction transmission electron microscopy (TEM) investigations were carried out using a Field Emission Transmission Electron Microscope (JEM-F200, JEOL Ltd., Japan) at an accelerating voltage of 200 kV.

## 3. Results

### 3.1. Morphology of the Nitrided Case

**Figure 1:**
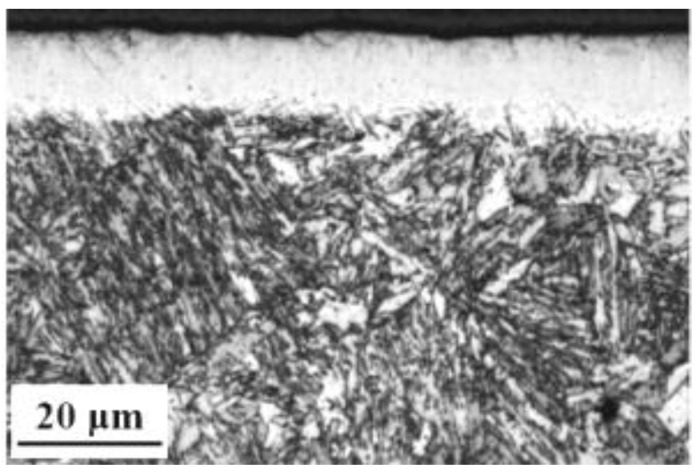
Micrograph of cross-sections over the nitrided layer of 17-4 martensitic stainless steel.

Figure 1 displays the cross-sectional microstructure of the 17-4 martensitic stainless steel after nitriding. The process produced a continuous, dense surface layer with high structural integrity. This nitrided zone exhibits a well-defined interface with the substrate, though its thickness varies anisotropically between approximately 11 and 14 μm. Under optical microscopy, the layer appears as a bright white band, a characteristic often associated with enhanced corrosion resistance due to the formation of nitrogen-saturated phases, such as expanded austenite [12]. Below this modified region, the substrate retains the typical lath martensitic structure of the untreated 17-4 steel, indicating that the martensitic matrix remains thermally stable under the present nitriding conditions at 350 °C.

### 3.2. Depth Profile of Nitrogen Concentration and Hardness

**Figure 2:**
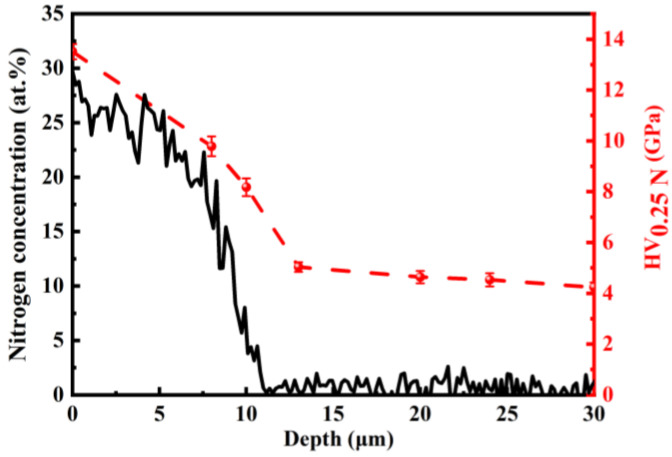
Nitrogen concentration-depth and hardness-depth profiles over the nitrided layer on 17-4 martensitic stainless steel.

Figure 2 depicts the depth profiles of nitrogen concentration and hardness (black and red lines, respectively) within the nitrided layers of 17-4 PH stainless steel. The surface nitrogen concentration reaches a maximum of 29.7 at.% and gradually decreases over the first 8 μm. Beyond this depth, a sharp drop in nitrogen content occurs toward the diffusion front at about 11 μm. Such a profile is characteristic of low-temperature nitriding in Fe-based alloys containing strong nitride-forming elements like chromium [9]. The abrupt decrease in nitrogen concentration can be attributed to the strong trapping of nitrogen interstitials by chromium atoms. This trapping promotes the formation of Cr-N short-range ordering (SRO) and significantly retards further nitrogen diffusion [13,14]. As a result, a steep concentration gradient develops near the advancing diffusion front, which is consistently reported in chromium-containing ferrous systems [15].

The hardness profile exhibited a strong correlation with the nitrogen concentration distribution, confirming that the mechanical properties of the modified surface layer are directly governed by its chemical composition. At the immediate surface, where nitrogen concentration is highest, a hardness value of HV_0.25N_ 13.5 GPa was measured, significantly exceeding that of the untreated substrate. With increasing depth, hardness gradually decreased, approaching substrate-equivalent levels at approximately 13 μm below the surface and stabilizing thereafter. This progressive reduction in hardness aligns consistently with the declining nitrogen gradient, establishing a clear composition–property relationship within the nitride layer.

### 3.3. X-Ray Diffraction Analysis

**Figure 3:**
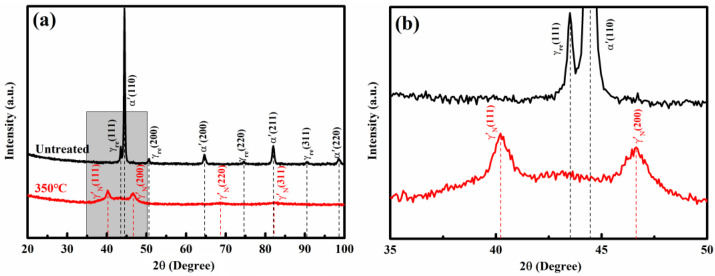
XRD patterns of the untreated 17-4 martensitic stainless steel are shown by the black line, and the nitrogen-modified layers on 17-4 martensitic stainless steel are shown by the red line, (**a**) the full diffraction pattern, (**b**) the magnified diffraction pattern correspond to the grey area in (**a**).

Figure 3 displays the X-ray diffractograms of the nitrided layers and the untreated 17-4 martensitic stainless steel, shown as red and dark lines, respectively. The base steel is composed primarily of martensite with a minor fraction of retained austenite. Its corresponding body-centered cubic (BCC) reflections are sharp and well-defined, indicative of a highly crystalline initial structure with low lattice strain and minimal defect density. Following nitriding, a new set of significantly weakened and broadened face-centered cubic (FCC) diffraction peaks emerged. While the (111) and (200) peaks remain clearly visible, the (220) and (311) signals become scarcely detectable, and the (222) peak vanishes entirely, as shown in Figure 3a.

The overall peak positions align with those typical of nitrogen-expanded austenite obtained from nitrided austenitic stainless steel. However, the marked reduction in intensity and pronounced peak broadening suggest a substantially higher defect density or refined grain size. Furthermore, a broad diffuse hump appears beneath the broadened (111) and (200) FCC peaks in Figure 3b. This hump-like scattering is characteristic of structural disorder, often associated with the formation of amorphous or ultrafine nanocrystalline phases under non-equilibrium processing conditions. Similar diffraction features are frequently reported in studies on severe plastic deformation techniques, such as ball milling [16]. Thus, both severe plastic deformation and interstitial supersaturation may share underlying mechanisms in driving microstructural refinement and amorphization.

### 3.4. TEM Characterization of Cross-Sections

**Figure 4:**
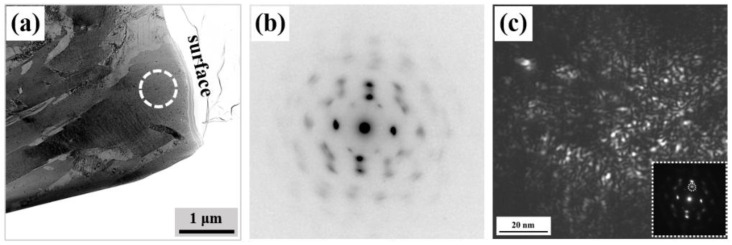
TEM characterization of low-temperature nitrided layers on 17-4 martensitic stainless steel. (**a**) The bright-field micrograph. (**b**) The selected area showing electron diffraction (SAED) patterns. (**c**) The dark-field image.

To further elucidate the microstructure of the nitrided surface layer, cross-sectional specimens of the nitrided 17-4 steel were examined using transmission electron microscopy (TEM). Figure 4a presents a bright-field TEM micrograph of the modified layer, revealing a fine lamellar morphology that aligns with the original martensitic structure. The retention of this morphology indicates that the phase transformation did not proceed via a conventional austenitic transformation, which typically disrupts or reconfigures the martensitic plates through diffusion-controlled processes or recrystallization, leading to the formation of equiaxed austenitic grains.

During TEM analysis, crystallographic orientation mapping based on Kikuchi line diffraction was attempted. However, no clear or interpretable Kikuchi patterns could be acquired from the modified layer. This is attributed to an exceptionally high density of crystal defects, which introduce severe lattice distortions and disrupt the long-range periodicity required for coherent electron diffraction. Consequently, the strong interference with diffraction conditions prevented reliable orientation calibration.

Selected area electron diffraction (SAED) was conducted on the same region to examine the crystallographic characteristics of the nitrided layer. As shown in Figure 4b, a distinct array of diffraction spots with complex geometric arrangements was observed. The pattern does not correspond to a single-crystal orientation but comprises multiple sets of diffraction spots originating from differently oriented grains. Dark-field imaging was subsequently performed using prominent diffraction spots to correlate crystallographic information with microstructural features. The resulting images (Figure 4c) reveal that the bright regions, visible under specific diffraction conditions, correspond to nanoscale grains of about 3 nm. These are direct evidence of nanocrystallization within the nitrided layer. Such a nanocrystalline microstructure is known to enhance mechanical properties via grain refinement, consistent with the Hall–Petch relationship [17].

**Figure 5:**
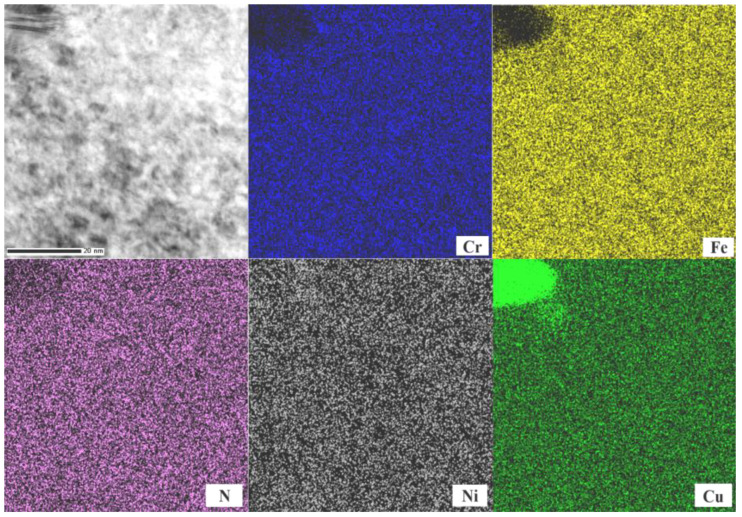
EDX analysis of the nitrided layer for different elements.

To determine whether the nanostructures were formed via a nondiffusion austenitic transformation rather than a diffusion-controlled precipitation mechanism, localized compositional analyses were systematically performed. Elemental distribution maps obtained by energy-dispersive X-ray spectroscopy (EDX) revealed the spatial partitioning of Fe, Cr, Ni, Cu, and N within the nitrided layer (Figure 5). Copper is predominantly confined to preexisting precipitates in the martensitic matrix, which are notably coarser than the nanostructures formed during nitriding. Importantly, no elemental segregation was observed within the nanostructured regions. This type of segregation, often resulting from diffusion-induced phase transitions, is typically associated with the formation of CrN and concomitant enrichment of Cr and N. These results collectively demonstrate that the nanostructures originate from a nondiffusion austenitic transformation, not a precipitation-mediated process. The spatially segregated Cu-rich particles further confirm that copper precipitation does not participate in the nanocrystallization mechanism during nitriding.

This nondiffusion phase transformation retains the original elements in solid solution, preserving their role in chemical processes, particularly corrosion resistance. By contrast, diffusion-controlled phase transitions typically induce CrN precipitation, which depletes chromium from the matrix and degrades passivation capability, thereby impairing corrosion resistance [12]. Avoiding such detrimental effects has been a key driver for the development of low-temperature nitriding technologies.

## 4. Discussion

Low-temperature nitriding represents a promising surface engineering strategy for enhancing the hardness and wear resistance of martensitic steels. This process typically enriches the near-surface region with interstitial nitrogen, yet the microstructural evolution at the diffusion front under low-temperature constraints (typically <450 °C) remains insufficiently elucidated. Under such low-temperature conditions, the bulk diffusion of iron and substitutional alloying elements (e.g., Cr) is severely suppressed, thereby inhibiting conventional nucleation-and-growth phase transformations [18]. This study provides experimental evidence that low-temperature nitriding induces nanocrystallization in martensitic steel via a unique diffusion-controlled mechanism. This process is governed by the interstitial influx of nitrogen, which triggers a localized martensite-to-austenite phase transformation. As nitrogen atoms diffuse inward from the surface, a steep concentration gradient develops at the advancing diffusion front. Notably, the low temperature limits long-range diffusive rearrangement of metal atoms, while thermodynamically, the increasing nitrogen concentration stabilizes the FCC austenite phase [19]. To accommodate this driving force under frozen diffusive kinetics, the system undergoes a diffusionless, military transformation involving coordinated atomic strain [20].

At the diffusion front, two forms of energy accumulate synergistically: (i) chemical-free energy (ΔG_chem_), arising from the enhanced thermodynamic stability of austenite with increasing nitrogen content, and (ii) elastic-strain energy (ΔG_strain_), generated by severe lattice distortion due to interstitial nitrogen in the BCC martensite. The total free energy change (ΔG_total_ = ΔG_chem_ + ΔG_strain_) rises with local nitrogen concentration. Once a critical concentration, and thus a critical ΔG_total_, is reached, the energy barrier for phase transformation is overcome, triggering an instantaneous strain-type transition from BCC martensite to FCC austenite.

**Figure 6:**
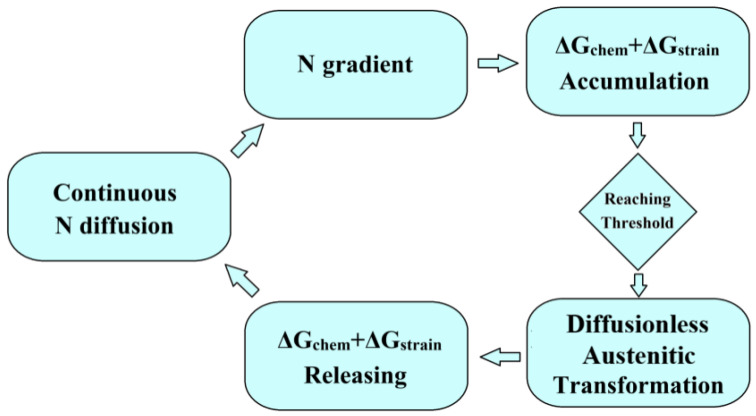
Schematic diagram of dynamic cycle for diffusionless austenitic transformation.

This transformation is extremely rapid, nearly approaching the speed of sound [21], and is characterized by an abrupt energy release: accumulated elastic-strain energy and chemical-free energy are relieved upon formation of the close-packed austenite lattice and partially converted into latent heat and interface energy in the high-density nano-grain boundaries [22]. Microscopically, each event corresponds to a discrete transformation burst. The high nitrogen concentration gradient results in an extremely narrow region at the diffusion front that satisfies the phase transformation criteria, reaching the nanoscale. Consequently, each phase transformation event leads to the formation of partially nanostructured domains. However, as nitrogen diffusion continues, the cycle repeats as follows: behind the transformed zone, nitrogen concentration is partially consumed or redistributed; ahead of it, fresh nitrogen influx renews the accumulation of chemical and strain energy. Once the critical threshold is reached again, another transformation occurs. Consequently, a periodic, self-sustaining dynamic cycle propagates along the diffusion front, as schematically illustrated in Figure 6.

In summary, the low-temperature nitriding of martensitic stainless steel is a non-equilibrium kinetic process characterized by strong coupling between two principal factors: (1) a diffusion-controlled nitrogen concentration field that changes the phase stability described by thermodynamic models, and (2) a diffusion-controlled stress/strain field that accumulates strain energy, driving phase transformation. The advancing diffusion front proceeds periodic accumulation of chemical driving force and localized elastic-strain energy. Once a critical threshold is reached, a diffusionless austenitic transformation is triggered on the nanoscale. This transformation releases energy abruptly, while continued nitrogen diffusion reinstates the conditions necessary for the next cycle, establishing a self-sustaining periodic process, and leading to layer-by-layer transformation and nanocrystallization.

## 5. Conclusions

This work experimentally confirmed a nanocrystallization pathway by low-temperature nitriding 17-4 martensitic stainless steel and provides a mechanistic framework for designing surface-nanostructured steels via controlled thermochemical processing. Low-temperature nitriding of martensitic stainless steel represents a non-equilibrium kinetic process driven by the coupling of two diffusion-controlled fields of chemical and elastic. At the diffusion front, the cyclic accumulation of chemical and elastic energy reaches a critical threshold, triggering a diffusionless austenitic transformation. The released energy and continued diffusion create a self-sustaining periodic mechanism, resulting in sequential layer-by-layer transformation and nanocrystallization.

## Figures and Tables

**Table 1:** Chemical composition of 17-4PH martensitic stainless steel (in wt.%).

C	Si	Mn	Cr	Ni	Cu	Nb	P	S	Fe
≤0.07	0.40	0.82	16.03	4.46	4.25	0.32	≤0.022	≤0.010	bal.

## Data Availability

The original contributions presented in this study are included in the article. Further inquiries can be directed to the corresponding author.
